# Compassionate Conservation Clashes With Conservation Biology: Should Empathy, Compassion, and Deontological Moral Principles Drive Conservation Practice?

**DOI:** 10.3389/fpsyg.2020.01139

**Published:** 2020-05-27

**Authors:** Andrea S. Griffin, Alex Callen, Kaya Klop-Toker, Robert J. Scanlon, Matt W. Hayward

**Affiliations:** ^1^Animal Behaviour and Cognition Lab, School of Psychology, University of Newcastle, Callaghan, NSW, Australia; ^2^Conservation Biology Research Group, School of Environmental and Life Sciences, University of Newcastle, Callaghan, NSW, Australia

**Keywords:** compassionate conservation, empathy, compassion, ethical bias, conservation decision making

## Abstract

“Compassionate Conservation” is an emerging movement within conservation science that is gaining attention through its promotion of “ethical” conservation practices that place empathy and compassion and the moral principles of “first, do no harm” and “individuals matter” at the forefront of conservation practice. We have articulated elsewhere how Compassionate Conservation, if adopted, could be more harmful for native biodiversity than any other conservation action implemented thus far, while also causing more net harm to individuals than it aims to stop. Here, we examine whether empathy, compassion and inflexible adherence to moral principles form a solid basis upon which to meet the goals of conservation biology as specified by pioneers in the discipline. Specifically, we examine a large empirical literature demonstrating that empathy is subject to significant biases and that inflexible adherence to moral rules can result in a “do nothing” approach. In light of this literature, we argue that our emotional systems have not evolved to provide a reliable basis for making decisions as to how best to ensure the long-term persistence of our planet. Consequently, in its most radical form, the Compassionate Conservation philosophy should not be enshrined as a legalized guiding principle for conservation action.

## Introduction

Conservation science is concerned with phenomena that affect the maintenance, loss, and restoration of biodiversity and the science of sustaining evolutionary processes that engender genetic, population, species and ecosystem diversity (Groom et al., 2006; Hunter and Gibbs, 2006; Van Dyke, 2008; Sahney et al., 2010). The need for conservation science stems from estimates suggesting that up to 50% of all species on the planet will disappear within the next 50 years (Koh et al., 2004), which will contribute to poverty, starvation, and will reset the course of evolution on this planet (Jackson, 2008; Millenium Ecosystem Assessment, 2009). One of the founders of conservation science, Michael Soulé (1985) described five guiding principles for the field: (1) diversity should be preserved, (2) untimely extinctions should be prevented, (3) ecological complexity should be maintained, (4) evolutionary processes should continue, and (5) biological diversity has intrinsic value (Lacy, 1993; Possingham and Davies, 1995; Christensen et al., 2009; Kareiva and Marvier, 2012; De Leenheer, 2014). Hence, preserving the diversity of life is a core tenet of conservation.

One can ask why conservation biology places so much emphasis on maintaining biodiversity. Soulé’s first four principles can be justified on the basis of utilitarian arguments. First, biodiversity provides humans with numerous health, social, spiritual and economic returns, as well as potential unpredictable opportunities for future innovations in all these domains (WHO, 2016). Second, due to the interdependent nature of all living beings, biodiversity sustains ecosystem function, which in turn sustains life. It is important to point out that, under these utilitarian arguments, the conservation of species is not really a goal in itself, but an instrument for achieving a goal. Soulé’s last principle is the only one to allude to the intrinsic value of biodiversity and its right to be conserved over and above its utilitarian benefit to humans.

To meet the goal of maintaining biodiversity, conservation practice requires on-the-ground actions. Decisions of what conservation management actions to implement were originally based on the gut feel of conservation practitioners. Scientific evidence has begun to take precedence, however. To this effect, practitioners have developed a suite of optimal decision-making tools that minimize subjective human influences, such as project prioritization protocols (Joseph et al., 2009), protected area placement (Christensen et al., 2009; De Leenheer, 2014) and species management (Lacy, 1993; Possingham and Davies, 1995). The over-arching aim of these frameworks is to ensure that best practice is evidence-based, where evidence pertains to the effectiveness with which a conservation action is in line with the stated goals of conservation biology.

These decision-making structures are being challenged by the recent emergence of a compassionate conservation movement, which argues that conservation actions should be founded first and foremost on empathy and compassion (Bekoff, 2013; Ramp and Bekoff, 2015; Wallach et al., 2018). By founding conservation practice on moral rules including “first, do no harm” and “individuals matter” (Wallach et al., 2018), compassionate conservation suggests shifting decision making in conservation biology from a utility-based practice to a moral-based practice. While utilitarian considerations determine whether an action is appropriate based on whether it maximizes a given utility for all, moral considerations examine whether an action is right based on whether stated moral principles are respected (McConnell, 2018). Endorsing such a paradigm shift requires a closer examination of whether using morality as a foundation for conservation actions is compatible with the over-arching goals of conservation biology, that is, to restore the perturbed and dysfunctional ecosystems that currently support a fraction of biodiversity that existed even just a few centuries ago (Ceballos et al., 2015).

In this article, we address two questions. Drawing upon a large body of empirical work on empathy and compassion by psychologists, we ask whether these capacities form a solid foundation on which to build conservation practice. Then, we examine closely the particularly controversial issue of species lethal control, which we re-frame in the light of an extensive body of psychological research on moral reasoning. Similar to our exploration of empathy, we address the question of whether principles such as “first do no harm” and “individuals matter” form solid foundations for sound decision making in conservation practice. We conclude by highlighting what we see as an important distinction between energizing public engagement and enshrining compassionate conservation moral principles into environmental law.

## Empathy and Compassion and Their Potential Role in Conservation Decision Making

A central tenet of compassionate conservation is that empathy should form the founding principle for conservation action (Vucetich and Nelson, 2013). Although the motivations for making empathy a cornerstone of conservation practice are possibly diverse, one identifiable origin is in a proposed return to virtue ethics to address the “depraved morality that Utilitarianism offers” (Cafaro, 2001; Vucetich and Nelson, 2013). Virtue ethics are a very ancient approach to defining how humans ought to act. In virtue ethics, morality stems from the character of an individual, rather than being a reflection of the actions of that individual (Hursthouse and Pettigrove, 2018). For example, in the time of ancient Greece, Aristotle defined the purpose of human life as living well, which is, living according to reason. Bravery, generosity, temperance, and magnanimity were considered manifestations of that purpose (Kraut, 2018). Honesty, courage, compassion, generosity, tolerance, love, fidelity, integrity, fairness, self-control, and prudence are all examples of virtues. Vucetich and Nelson (2013) have proposed that in the context of conservation, “the purpose of a person living a sustainable life would have to be “*to treat others as one would be treated, if one were in their position.*” It is here that empathy plays a crucial role. Indeed, empathy is taken to provide the “objective, empirical knowledge …about the conditions and capacities of others (to flourish and suffer)” (Vucetich and Nelson, 2013).

Empathy has a long and strong study tradition amongst psychologists. One use of the term refers to the capacity to feel what you infer others are feeling (de Vignemont and Singer, 2006; Singer and Klimecki, 2014; Bloom, 2017a). Within this view, *empathy*, also referred to as *affective empathy*, *emotional empathy*, *experience sharing*, or *personal distress* (Zaki and Ochsner, 2011; Bloom, 2017b; Zaki, 2017), is a form of shared affective arousal (e.g., sadness), which is triggered automatically and involuntarily, although people can retain agency in how closely they subsequently align their empathic reactions to their goals (Zaki, 2018). Empathy thus defined is distinguished conceptually and empirically from other aspects of social cognition, including, for example, people’s interest in taking, and understanding of, the perspectives of others (referred to as “cognitive empathy”) and *fantasy*, specifically, the tendency to identify with fictional characters (Davis, 1983; Decety and Jackson, 2004; Eisenberg and Eggum, 2009; Jordan et al., 2016). Other researchers view empathy as a multi-dimensional construct encompassing affective, cognitive and emotion regulation dimensions (Decety, 2011; Eres and Molenberghs, 2013; Baldner and McGinley, 2014). Taking an evolutionary perspective with the aim of emphasizing phylogenetic continuity and adaptive significance, Preston and de Waal (2002) argue that their Perception-Action model of empathy recoheres discrepant views into a unified whole. The model refers to the object as the primary individual who experiences an emotion or state and to the subject as the individual who secondarily experiences or understands the emotion/state of the object. Empathy is then defined as “*any* < italics added by the present authors > process where the attended perception of the object’s state generates a state in the subject that is more applicable to the object’s state than to the subject’s prior state or situation.” Yet, what should and should not be included under the term empathy remains a matter of debate (Bloom, 2017a; Zaki, 2017).

Over and above the debate on how exactly to define and measure empathy, there is significant support for the hypothesis that empathy predicts prosocial behavior (Batson et al., 1981; Batson, 1998, 2016). As a result, there are many advocates of the idea that empathy can be used as a moral compass. The issue of significant concern here is that an extensive body of work has now demonstrated that our empathic responses are fraught with biases (Fetherstonhaugh et al., 1997; Kogut and Ritov, 2005; Slovic, 2007; Cikara et al., 2011). The biased nature of our empathetic responses has caused scholars of empathy to urge for the creation and commitment to institutional, legal, and political systems that draw upon reasoned analysis and not empathy (Slovic, 2007; Bloom, 2011, 2017a; Slovic et al., 2011; Västfjäll et al., 2017; Zaki, 2017). These calls cast serious doubt on whether our empathetic responses are the best tool to replace the utilitarian based principles that currently underpin conservation actions on the ground. We discuss these biases below.

The first bias of empathy that may interfere with sound conservation decisions is that empathy favors the familiar and the in-group (reviewed by Cikara et al., 2011), and new research is beginning to show that these preferential responses have distinct neural signatures (Xu et al., 2009; Eres and Molenberghs, 2013). For example, work examining the relationship between the willingness to help and the number of humans in need of help has revealed that an identified single victim elicits considerably more assistance than a non−identified single victim (Kogut and Ritov, 2005; Västfjäll et al., 2014). Willingness to help varies with the type of identifying information, however, people contribute more toward individuals identified with a picture than individuals identified only by age (Kogut and Ritov, 2005). These findings are in line with the more general finding that people are more generous toward an identifiable victim than toward a statistical victim (Slovic, 2007; Small et al., 2007a). With regards to in-group biases, research has shown, for example, that people are more likely to help individuals to whom they are genetically related (Burnstein et al., 1994) and with whom they share a nationality (Levine and Thompson, 2004). Applied to conservation practice, these familiarity and in-group biases mean that species we are familiar with and species that resemble us are likely to elicit more empathy than those we are unfamiliar with and dissimilar to. As a result, conservation actions involving these species are likely to seem more morally justified independent of whether that action serves to restore/maintain biodiversity. Compassionate conservationists who advocate for empathy to become a guiding principle of conservation practice restrict their compassion almost entirely to large, charismatic mammals (Hayward et al., 2019).

The second bias of empathy that raises concerns over its use as a foundational moral principle in conservation science is that empathy does not scale. That is, people are deeply moved by one person’s suffering but remain affectively untouched by large-scale losses of human life (Fetherstonhaugh et al., 1997; Slovic, 2007; Slovic et al., 2011; Västfjäll et al., 2014). Humans can experience both psychic numbing, which is a reduced emotional sensitivity to shocking and emotionally overwhelming experiences (Lifton, 1967; Slovic, 2007; Slovic et al., 2011; Dickert et al., 2012) and psychological numbing that consists of a cognitive and perceptual form of insensitivity that reduces our ability to evaluate the consequences of our actions (Fetherstonhaugh et al., 1997). Kogut and Ritov (2005) found that people tend to report feeling more distress and compassion when considering a single identified victim than when considering a group of victims, even if identified. We also know that people put more weight on the proportion of lives saved than on the number of lives saved even when the number of lives saved is the same or even less (Slovic, 2007). Fetherstonhaugh et al. (1997) showed that people are less willing to send help that would save 4,500 lives in Rwandan refugee camps as the size of the camps’ at-risk population increased. This is because saving 80% of 100 lives elicits a greater affective response than saving 20% of 1,000. The failure of our emotional systems to track the suffering of large-scale atrocities is invoked to explain the indifference with which many of the 20th century mass murders were treated (Slovic et al., 2011; Västfjäll et al., 2014). By the same token, psychological and psychic numbing are likely to be adaptive. Research has shown that individuals scoring high on empathy are more prone to depression and anxiety, that is, excessive empathy leads to burn-out (Schreiter et al., 2013).

Biases in empathy that favor small numbers of identifiable individuals might potentially arise because we process information related to individuals in fundamentally different ways to which we process information on groups of individuals. As Bloom (2017a) puts it “Empathy resonates to the suffering of identifiable victims but is largely silent when it comes to both future costs and statistical benefits.” Our empathic responses likely served us well to protect individuals and their small family and community groups from present, visible, immediate dangers, but they did not evolve to help us respond to distant calamities. As a result, researchers have argued that “deliberate mechanisms are needed to counteract the innumeracy and parochialism of empathy” (Västfjäll et al., 2017), and over and above any discrepancy in the definition of empathy, scholars mostly agree that affective empathetical responses should not be used as a guide to social policy, legal systems (Bloom, 2017a, b) and large-scale collective actions of organizations and nations (Västfjäll et al., 2017; Zaki, 2017). These calls for caution are equally applicable to conservation actions, which should involve careful consideration of statistical and predictive information to guide evidence-based decision making (Urban et al., 2016).

In addition to empathy, compassionate conservation, as the name indicates, asserts that compassion should also become a moral foundation of conservation practice. One problem is that the study of compassion is fraught with even greater terminological debate than empathy – so much so that some consider compassion to be an equally poor guide when assessing what is right and wrong (Västfjäll et al., 2017). For example, some define compassion as “feeling sorrow or concern for the suffering of another person, coupled with the desire to alleviate that suffering” (Keltner et al., 2014), which seems to give compassion the same affective component as empathy. Other scholars of empathy and compassion distinguish between the two by referring to feeling *for* (compassion) vs. feeling *with* (empathy) the other (Singer and Klimecki, 2014). In this framework, empathy – the capacity to feel what one infers others are feeling – is separate from compassion, which involves feelings of warmth, concern and care for others with a strong motivation to help (Singer and Klimecki, 2014). In line with this framework, qualitative research in palliative care suggests that empathy and compassion are distinguishable and are experienced differently by terminally ill patients (Sinclair et al., 2017). Gilbert (2016) has defined compassion as “a sensitivity to suffering in self and in others with a commitment to try to alleviate and prevent it,” a definition that brings to the fore the motivation to prevent suffering in the self and others. In a recent review of definitions and controversies, however, Gilbert (2017) reports that compassion has many textures and definitions and it would be unwise to settle on certain definitions without a better understanding of the processes that underpin compassion. Some have argued that the definition of compassion remains too vague to be sure compassion is not biased in the very same way as empathy (Västfjäll et al., 2017). For example, it has been argued that studies investigating biases in empathy are actually biases in compassion. Self-report and psychophysiological data indicate that people’s bias toward an identifiable victim (Small et al., 2007b) and single individuals (Västfjäll et al., 2014) are not driven by empathy but rather a loss of compassion (Västfjäll et al., 2014, 2017).

There is an increasing interest in training humans to experience compassion (Klimecki et al., 2013; Weng et al., 2013; Chierchia and Singer, 2017) and a recent systematic review has found small to medium effects on self-reported emotions and observed behavioral outcomes (Luberto et al., 2018). Whether compassion training generates enduring states of being that are free from the biases of empathy and have a smaller risk of apathy is not yet known. Skilled Buddhist monks trained in love and compassion meditation practices for decades hardly seem like a reasonable working model for compassion training in the broader population. Further, if compassion is distinct from empathy but requires large-scale training in the general population, then the breadth and the time lag hardly seem suited to addressing the urgency of the biodiversity crisis.

As Slovic and Västfjäll (2010) put it: “Left to its own devices, moral intuition will likely favor individual victims and sensational stories that are close to home and easy to imagine. Our sizable capacity to care for others may be demotivated by negative feelings resulting from thinking about those we cannot help, or it may be overridden by pressing personal and local interests. Compassion for others has been characterized by social psychologist Daniel Batson as “a fragile flower, easily crushed by self-concern” (Batson, 1983, 1990). Faced with genocide and other mass tragedies, we cannot rely on our moral intuitions to guide us to act properly. All too often, these intuitions seduce us into calmly turning away from massive losses of human lives, when we should be driven by outrage to act. This is no small weakness in our moral compass.”

Outpourings of support for individually identified animals, coupled with the frequent indifference of humans toward the destruction of natural habitats suggest that our empathy toward non-humans is fraught with the same biases as our empathy toward humans (Macdonald et al., 2016; Levin et al., 2017; Buhrmester et al., 2018). Gross errors of our empathic responses can be found in our obsession with saving injured wildlife, even if it condemns them to a life in a cage or is ultimately ineffectual (Augee et al., 1996; Sharp, 1996), and feeding wildlife in ways that cause disease and suffering (Bryant, 1994; Orams, 2002). The narrow-minded and innumerate quality of our empathetic responses make them fundamentally ill-tuned to determining whether a conservation action that seeks to safeguard biodiversity is justified or not. Compassion toward non-humans, just like our compassion toward humans, in so far that we even know what compassion is, might well be fraught with the same biases as our empathetical responses and provides no better avenue. To replace reason-based principles with principles that draw upon our empathic responses to living creatures is to formalize, legalize, and solidify our evolutionary biases into decision-making structures. It is likely that evidence of these biases can already be found in conservation practice (Heeren et al., 2017) but based on what we know about our empathic systems, we should be seeking to reduce the influence of empathy, not enhance it.

## The Role of Moral Judgements and Moral Dilemmas in Species Lethal Control

Of central concern to those who adhere to the ideas of compassionate conservation is the killing of introduced species as a means to restore and manage ecosystems. For example, rabbits (*Oryctolagus cuniculus*), red foxes (*Vulpes vulpes*), feral cats (*Felis catus*) and common mynas (*Acridotheres tristis*), a songbird, are species introduced to Australia by European colonialists within the last 200 years that are lethally managed across large areas of the country (Mahon, 2009). Lethal control is not limited to introduced species, however, as is evident in management of native songbirds (e.g., noisy miners *Manorina melanocephala*), flying foxes *Pteropus spp.*, and kangaroos *Macropus spp.* (Descovich et al., 2016; Florens, 2016; Beggs et al., 2019). Compassionate conservationists have repeatedly reiterated their opposition to such practices based on the moral principles “individuals matter” and “first, do no harm” (Bekoff, 2007; Wallach et al., 2018). The conservation practice of killing non-human animals violates legitimate values of life that place the emphasis on individual-level animal welfare.

The problem of killing invasive animals can be viewed through the lens of a moral dilemma. Moral dilemmas arise when two moral rules come into conflict, for example, the moral duty to help vs. the moral duty not to harm. Viewed through the lens of a moral dilemma, lethal control of introduced species creates conflict between adhering to the moral duty to save those animals at risk of extinction, and a moral aversion to inflicting harm on living creatures.

How people resolve moral dilemmas is heavily researched in the fields of moral psychology and moral cognition, and increasingly in the neurosciences (Greene et al., 2001; Christensen and Gomila, 2012; Conway and Gawronski, 2013). The most common experimental paradigm is the “trolley dilemma,” which involves a runaway trolley that is heading for five railway workers who will be killed if the trolley continues its course. Participants are asked to take the perspective of a character in the scenario who can choose to leap in and to pull a switch to redirect the trolley onto a different track and save the five railway workers. However, redirecting the trolley on to an alternative track will kill one railway worker who would otherwise not have been killed. The “action” that the character in the scenario can choose to carry out, or to not carry out, is referred to as a moral transgression whereas the choice between the act of committing or omitting to carry out the moral transgression is termed a moral judgment. Other experimental paradigms involve global epidemics, terrorist attacks, desperate survivors on lifeboats, and speeding trains.

Sacrificial dilemmas are used with the view that they provide a useful contrast between utilitarian and deontological principles (Singer, 2005; Greene et al., 2008; Christensen and Gomila, 2012; Conway and Gawronski, 2013; but see Kahane, 2015). Deontology is an ethical theory that advocates the use of rules to distinguish right from wrong actions, whereas utilitarianism is a form of consequentialism, an ethical theory that judges the right or wrongness of actions by their results. The principle of deontology posits that the morality of an action is determined by the inherent nature of the action, while the principle of utilitarianism posits that the morality of an action is determined by its consequences. For example, while inflexible adherence to deontological principles asserts that lying is wrong, consequentialism results in the view that lying is right if it saves someone’s life (Schermer, 2007). In a sacrificial dilemma, the decision to commit the harm is referred to as a utilitarian moral judgment, because it weighs costs and benefits (sacrifice one to save a greater number), while the decision to refrain from doing harm, and in the context of such dilemmas “do nothing,” is a deontological moral judgment (it is wrong to do so), because it gives more weight to the “do not kill” principle. In the former, aggregate welfare is maximized, while in the latter, adherence to moral rules takes precedence.

Considerable research has examined the psychological mechanisms that underpin dilemma responses (Christensen and Gomila, 2012). Although there is now evidence that affect and deliberative processing play some role in both utilitarian and deontological inclinations, evidence overwhelmingly supports the claim that deontological responses involve relatively more affective processing about harmful actions (Bartels, 2008; Conway and Gawronski, 2013; Conway et al., 2018), mediated by the sensorimotor representations of performing harm (Miller et al., 2014) and in generating harm in others (Christov-Moore et al., 2017). By contrast, utilitarian responses tend to involve relatively more deliberative reasoning about outcomes. For example, requiring participants to undertake another cognitive task while simultaneously making moral judgements makes them slower or less likely to select a utilitarian outcome, but does not affect deontological choices (Greene et al., 2008; Trémolière et al., 2012; Conway and Gawronski, 2013; Jeurissen et al., 2014), while increasing empathy enhances deontological inclinations without impacting utilitarian ones (Conway and Gawronski, 2013). Higher emotional arousal predicts deontological judgment (Szekely and Miu, 2015; Zhang et al., 2017) while lower self-regulation as indexed by resting heart rate variability is associated with utilitarian judgements (Park et al., 2016).

Deontological concerns are positively associated with empathic concerns, perspective taking and religiosity, while utilitarian concerns relate to the need for cognition and the ambition to reason extensively – a component of active, open-minded thinking (Conway and Gawronski, 2013; Szekely et al., 2015). People who make utilitarian judgments tend to score high on measures of reflective thinking vs. intuitive thinking (Bartels, 2008), working memory (Moore et al., 2008), and performance on the Cognitive Reflection Test, a measure of general reflective ability and open-minded thinking to some extent influenced by numeracy (Byrd and Conway, 2019). Positive reappraisal in which thoughts of attaching a positive meaning to negative events reduces deontological choices via its reducing effect on emotional arousal (Szekely and Miu, 2015).

Compassionate conservation’s strong stance against lethal control (Wallach et al., 2018) presents the hallmarks of a deontological resolution of the moral dilemma that lethal control creates. That is, faced with the moral dilemma of sacrificing a few to save the many, compassionate conservationists defer from harming anything and in so doing ultimately harm many more (Hayward et al., 2019; Callen et al., 2020). Compassionate conservationists do not want to kill because it is morally wrong to harm, that is, adherence to the moral rule takes precedence over the utilitarian benefit of saving many. This view is consistent with the emphasis of compassionate conservation on individual level welfare, potentially driven by a strong experience of single individual-oriented empathy (Gleichgerrcht and Young, 2013), which in turn acts as a strong motivator to avoid harm. As a result of the deontological moral choice, a “do nothing” approach is favored by compassionate conservationists over intervention. Indeed, compassionate conservationists have been vocal in expressing the view that humans should step back from managing the natural world, and “let nature take over” (Vucetich and Nelson, 2013; Marris, 2018; Wallach et al., 2018).

The deontological resolution of the moral dilemma also explains why compassionate conservation is immune to arguments that killing some animals improves animal welfare because ultimately it saves more animal lives. For example, when one fox is killed, there can be no doubt that the prey that fox would have eaten during its lifetime will be spared from predation by that fox (Callen et al., 2020). When one rabbit is killed, there can be no doubt that the vegetation it would have eaten remains available to less effective foragers who run less risk of starvation (Dawson and Ellis, 1994). Compassionate conservation is not open to such utilitarian reasoning because for compassionate conservationists, adherence to the moral rule “do not harm” always trumps any kind of utilitarian reasoning, even when it comes to individual-level animal welfare considerations. This inflexible stance is a constant source of frustration in heated exchanges amongst proponents and opponents of compassionate conservation because it results in inherent contradictions amongst compassionate conservations’ own principles. For example, if predation by foxes reaches levels where its prey species are reduced to the extent that they can no longer find a mate, or even cease to exist, or if rabbits consume so much vegetation that other animals starve, then the compassionate conservation principle of “first, do no harm” violates the principle of “individuals matter.” That violation cannot be resolved without some degree of utilitarian reasoning at the level of individual animal welfare.

## Conclusion

Much of the driving motivation behind advocates of compassionate conservation seems to originate in an experience of frustration with so-called numerous failures of conservation (Bekoff, 2013). Clearly, despite 40 years of work in the field of conservation science, natural habitats continue to be destroyed at rates never seen before and human activity remains the prevailing force behind the sixth mass extinction. Proponents of compassionate conservation seem convinced that this disastrous state of affairs provides evidence that the utilitarian, evidence-based decision-making frameworks that underpin conservation science have failed and should therefore be overhauled and replaced with empathy and the moral principles of “first, do no harm” and “individuals matter,” despite reviews finding conservation actions work (Hoffmann et al., 2010).

We argue that many of these failures are not failures of conservation science *per se*. Scientific principles remain the most powerful means of understanding and predicting the responses of natural systems (Urban et al., 2016). Most of the “failures” result not from the scientific method and the utilitarian principles that aim to restore and maintain biodiversity, but from the way in which conservation-related information is politically manipulated to precisely tap our emotional systems and drive scant resources toward causes toward which we feel more empathy and therefore greater moral duty to act. While some species can serve as a cause to indirectly protect habitat (Poiani et al., 2001; Roberge et al., 2008), many cases receive more attention than they deserve based on utilitarian principles (e.g., whales) precisely because they tap the evolutionary biases of our empathic systems (Small, 2011; Wallmo and Lew, 2012; Colléony et al., 2017). To attack conservation science on moral grounds using the argument that conservation failures prove that the scientific method is not working is to overlook the social and political complexities that lie at the heart of the current biodiversity crisis.

Empathy has its place in fostering pro-environmental attitudes amongst the general public in that it can act as a strong motivator of individual-level action (Eisenberg and Miller, 1987). The person who sees koalas burning in bushfires is suddenly sufficiently motivated to write to their local politician to demand action on climate change. This is good thing in our view and an appropriate application of empathy. Affect is a key component of decision making and without it, people are impaired in their capacity to make decisions (Damasio, 1994; Gupta et al., 2011; Jamil, 2014). But acknowledging and allowing space for affect to drive individual-level pro-environmental actions is a very distinct agenda from advocating that empathy and the deontological moral judgements empathy energizes should be formalized and legitimized into the political and legal structures that underpin conservation action and determine whether or not invasive animals can be controlled. For that agenda, we need to heed the concerns of the scholars of human behavior and avoid being attracted to intuitively appealing but elusive concepts like empathy and compassion, and their associated inflexible adherence to two moral rules.

## Author Contributions

AG wrote the manuscript with the assistance of KK-T. All authors contributed to the conception, design of the manuscript, literature overview, edited, and improved the manuscript.

## Conflict of Interest

The authors declare that the research was conducted in the absence of any commercial or financial relationships that could be construed as a potential conflict of interest.
